# Transcriptomic analysis identifies Toll‐like and Nod‐like pathways and necroptosis in pulmonary arterial hypertension

**DOI:** 10.1111/jcmm.15745

**Published:** 2020-08-29

**Authors:** Genfa Xiao, Wei Zhuang, Tingjun Wang, Guili Lian, Li Luo, Chaoyi Ye, Huajun Wang, Liangdi Xie

**Affiliations:** ^1^ Department of Geriatrics The First Affiliated Hospital of Fujian Medical University Fuzhou Fujian People’s Republic of China; ^2^ Department of General Medicine The First Affiliated Hospital of Fujian Medical University Fuzhou People’s Republic of China; ^3^ Fujian Hypertension Research Institute The First Affiliated Hospital of Fujian Medical University People’s Republic of China

**Keywords:** inflammation and immunity, necroptosis, Nod‐like receptor, pulmonary arterial hypertension, pulmonary vascular remodelling, RNA sequencing, Toll‐like receptor

## Abstract

Inflammation and immunity play a causal role in the pathogenesis of pulmonary vascular remodelling and pulmonary arterial hypertension (PAH). However, the pathways and mechanisms by which inflammation and immunity contribute to pulmonary vascular remodelling remain unknown. RNA sequencing was used to analyse the transcriptome in control and rats injected with monocrotaline (MCT) for various weeks. Using the transcriptional profiling of MCT‐induced PAH coupled with bioinformatics analysis, we clustered the differentially expressed genes (DEGs) and chose the increased expression patterns associated with inflammatory and immune response. We found the enrichment of Toll‐like receptor (TLR) and Nod‐like receptor (NLR) pathways and identified NF‐κB‐mediated inflammatory and immune profiling in MCT‐induced PAH. Pathway‐based data integration and visualization showed the dysregulated TLR and NLR pathways, including increased expression of TLR2 and NLRP3, and their downstream molecules. Further analysis revealed that the activation of TLR and NLR pathways was associated with up‐regulation of damage‐associated molecular patterns (DAMPs) and RIPK3‐mediated necroptosis was involved in the generation of DAMPs in MCT‐induced PAH. Collectively, we identify RIPK3‐mediated necroptosis and its triggered TLR and NLR pathways in the progression of pulmonary vascular remodelling, thus providing novel insights into the mechanisms underlying inflammation and immunity in the pathogenesis of PAH.

## 1 INTRODUCTION

Pulmonary arterial hypertension (PAH) is a devastating disease characterized by perivascular infiltration of inflammatory cells and pulmonary vascular remodelling, ultimately resulting in the right heart failure and premature death. Patient survival of advanced PAH remains poor,^1^ and the pathogenic mechanisms contributed to the progression of pulmonary vascular remodelling in PAH are not well understood.

It is widely accepted that inflammation and immunity are linked to pulmonary vascular remodelling in PAH.^2^ The infiltration of inflammatory cells, such as mast cells, macrophages, dendritic cells and lymphocytes, was identified in the PAH lung, and an array of inflammatory mediators, including TNFα, IL‐1β, IL‐6, IL‐8, IL‐12, MCP‐1 and RANTES, was abnormally elevated in peripheral blood.^2^ In addition, inflammatory infiltration was positively correlated with pulmonary arterial remodelling parameters.^3^ Although it is well established inflammation and immunity are involved in pulmonary vascular remodelling and pulmonary hypertension, the pathways and mechanisms by which inflammation and immunity contributed to pulmonary vascular remodelling remain unknown.

The monocrotaline (MCT) model of PAH was widely used for over 50 years.^4^ After administration of MCT, significant changes in pulmonary artery pressure, pulmonary arterioles remodelling and right ventricular hypertrophy occur.^5^ MCT was thought to induce a syndrome, composed of acute lung injury, necrotizing pulmonary arteritis in about one third of the animals and pulmonary hypertension, etc.^6^ The development of MCT‐induced PAH was associated with dysregulated inflammation/immunity, because inflammatory cells, mainly neutrophils, macrophages, dendritic cells and lymphocytes infiltrated the lung, mainly in perivascular areas.^4^ Consistently, our previous study has showed an elevated marker of macrophage infiltration. In addition to inflammatory cell infiltration, the inflammatory mediators, such as TNFα and IL‐6, were elevated, with concomitantly increased pulmonary arterial remodelling parameters WT% and WA% in the progression of MCT‐induced PAH.^7^

The transcriptomic change during the PAH progression was investigated by using microarray.^8^ Recently, high‐throughput RNA sequencing (RNA‐seq) has emerged as a more powerful alternative to microarray.^9^ We have performed RNA‐seq analysis of rat lungs isolated from control and monocrotaline (MCT)‐treated rats that had been treated with MCT for a variety of weeks, and this study showed that inflammatory and immune response was occurred at the early time‐point of PAH development and dysregulated inflammation/immunity were involved in the onset and progression of PAH.^10^

The changes of inflammation and immunity and pulmonary vascular remodelling that occur during the PAH progression largely result from the changes in the transcriptome. Therefore, in the current study, we use the RNA‐seq data set and bioinformatics approach to carry out a further analysis of the transcriptome in MCT‐induced PAH, aiming to have a deeper understanding of inflammatory and immune mechanisms in pulmonary vascular remodelling.

## 2 MATERIALS AND METHODS

### 2.1 Animal and treatment

All procedures have been conducted in accordance with the ARRIVE guidelines and were approved by the Laboratory Animal Welfare and Ethics Committee of Fujian Medical University (Approval No. 2017‐070, Fuzhou, China). Sprague‐Dawley rats (4 ‐ 5 weeks male and female rats, 200‐250g) were purchased from Shanghai SLACCAS Laboratory Animal Co., Ltd. (Certificate No. SCXK 2012‐0002). The rats were raised and housed in the animal room and received food and water ad libitum. PAH model in rats was induced by a single intraperitoneal injection of 40 mg/kg MCT (Sigma‐Aldrich) as described previously.^7^ A total of 17 rats were used in this study: 12 rats were randomly assigned into 4 groups and treated with MCT (n = 3, each group) and five remaining rats served as control and treated with saline. Before killing, an effort was made to diminish suffering by intraperitoneal injection of 30 mg/kg sodium pentobarbital. The MCT‐treated rats were killed at the end of weeks 1, 2, 3 and 4, and control rats were killed at week 0, and as with the corresponding MCT‐treated rats, at the end of weeks 1, 2, 3 and 4. Rat lungs were immediately isolated and frozen in liquid nitrogen and then stored at −80°C.

### 2.2 RNA extraction, cDNA library preparation and RNA‐seq

Total RNA was isolated from 50 mg lung tissues using 1 mL TRIzol reagent (Life Technology) following the manufacturer's instructions. RNA integrity and quality were assessed by gel electrophoresis, and its concentration and purity were determined by the Thermo Scientific NanoDropTM instruments. Total RNA with high quality was used for cDNA library preparation. Library preparation and RNA‐seq were performed on an Illumina HiSeq 2000 platform by Genergy Biotechnology (Shanghai) Co., Ltd. The generated raw sequences were processed through a series of steps: (a) removing the low quality reads and adapter sequences, (b) quality control using the FastQC software, (c) mapping the clean reads to rat reference genome using STAR software, (d) assembling transcripts using the software of StringTie and Cufflinks‐Cuffmerge, (e) calculation of transcripts abundance using FPKM, and (f) identification of DEGs using DESeq2 software.

### 2.3 Bioinformatics analysis

Bioinformatic analysis tools, including DAVID,^11^ KOBAS,^12^ Venny 2.1, GeneMANIA,^13^ Enrichr,^14^ Pathview,^15^ and Morpheus, were used in this study. Briefly, Hierarchical clustering analysis and heatmap creation were performed by Morpheus (https://software.broadinstitute.org/morpheus/). Gene Ontology (GO) enrichment analysis of clusters was performed by using DAVID (https://david.ncifcrf.gov/). KOBAS (http://kobas.cbi.pku.edu.cn/) was used for KEGG pathway enrichment analysis on the selected clusters. The overlapped inflammatory and immune genes in cluster 1 and cluster 3 of GO and KEGG pathway analysis were determined by Venny 2.1 (https://bioinfogp.cnb.csic.es/tools/venny/index.html). The genes annotated in pathways of TLR, NLR and necroptosis could be retrieved in KEGG PATHWAY Database (https://www.kegg.jp/kegg/pathway.html) by using the keywords of TLR, NLR and necroptosis pathway. GeneMANIA (http://bioinformatics.sdstate.edu/idep/) was used for analysis of the interaction among the overlapped genes. The modified Pathview (https://pathview.uncc.edu/home) was used for data integration and visualization of gene expression change in TLR, NLR and necroptosis pathways. Pathview analysis of gene expression change was modified by showing *P*‐values change instead of showing expression value ratio change. The enrichment of highly represented transcription factors was conducted by Enrichr (http://amp.pharm.mssm.edu/Enrichr/). The significant enrichment terms were determined by combined score, owing to the combination of *P*‐value and *z*‐score.

### 2.4 Real‐time PCR analysis

Total RNA was isolated from lung tissues of control and rats injected with MCT for 4 weeks. First‐strand cDNA was synthesized by using the Transcriptor First Strand cDNA Synthesis Kit, according to the manufacturer's protocol. Real‐time PCR was performed in accordance with the manufacturer's instructions, as previously described.^16^ The forward and reverse primers were synthesized by Sangon Biotechnology Co., Ltd. The rat origin primers were used for real‐time PCR analysis and listed as follows: forward—5’‐GTA GAC CTT AGA CGC GTA GG‐3’, and reverse—5’‐TAG GTG CTG AAG TGG CG TC‐3’, for RIPK1; forward—5’‐ GAG CGC GAC GCT AAT CGA G‐3’, and reverse—5’‐ CCT TTT CGC GCC AAG CAA TC‐3’, for XIAP; forward—5’‐ CAT TTT GTG GAC CCC AAG GC‐3’, and reverse—5’‐ GGC CCA TCT CAC TCA ACA GT‐3’, for CFLAR; forward—5’‐ GGA GCG CAG GAT AGA CCA AGG‐3’, and reverse—5’‐ CAC TGG TCA TAG ATG AGC TGG C‐3’, for MLKL; forward—5’‐ GTG ACC CTG AAG GAC AGT GG‐3’, and reverse—5’‐ TTG ATC AGG TGA GTC GTG CC‐3’, for TNFAIP3 (A20); forward—5’‐ ACT CTC AGC CGT AGA CGT TG‐3’, and reverse—5’‐ GAG AGA TCG ATG ACG CAC CA‐3’, for RIPK3; and forward—5’‐ TGC ACC ACC AAC TGC TTA GC ‐3’, and reverse—5’‐ GGC ATG GAC TGT GGT CAT GAG‐3’, for GAPDH. Quantification of gene mRNA expression was performed using Roche Real‐time PCR systems. The relative quantification was performed by the comparative 2^−ΔΔCT^ method and expressed as fold changes.

### 2.5 Statistical analysis

Morpheus software and R package software were used for RNA‐seq data set analysis. The DEGs between control and MCT treatment were identified by DESeq2 package in R using a threshold of fold‐change ≥2 and *P* ≤ .05. Data were shown as mean ± SEM, and comparison of two conditions in Pathview was performed by using Morpheus software. More details of the statistical analysis were provided in the figure legends.

## 3 RESULTS

### 3.1 Identification of pathways related to inflammation and immunity

**FIGURE 1:**
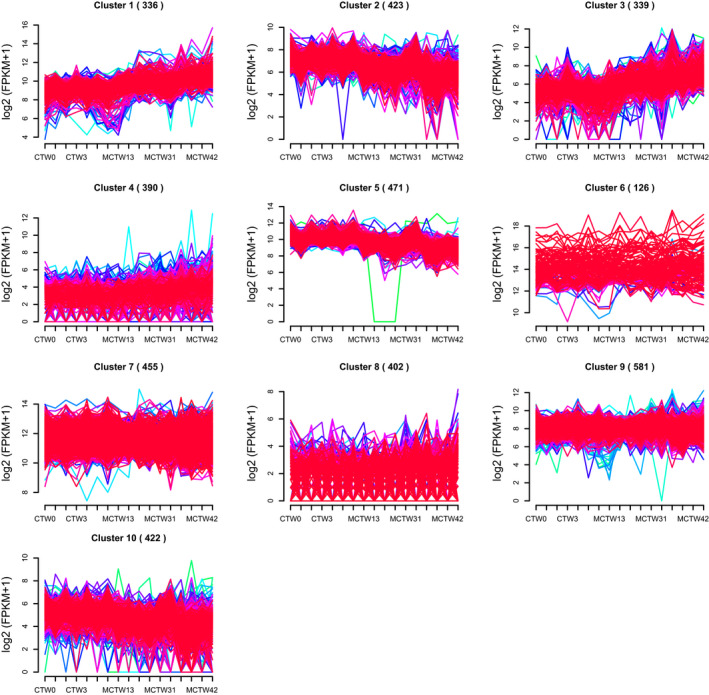
Clustering analysis of differentially expressed genes (DEGs). A total of 23 200 transcripts and 280, 1342, 908 and 3155 DEGs were identified at the end of weeks 1, 2, 3 and 4 after monocrotaline treatment. Hierarchical clustering of these DEGs generated 10 clusters of expression pattern (distance metric, Pearson correlation; linkage rule, average linkage)

**FIGURE 2:**
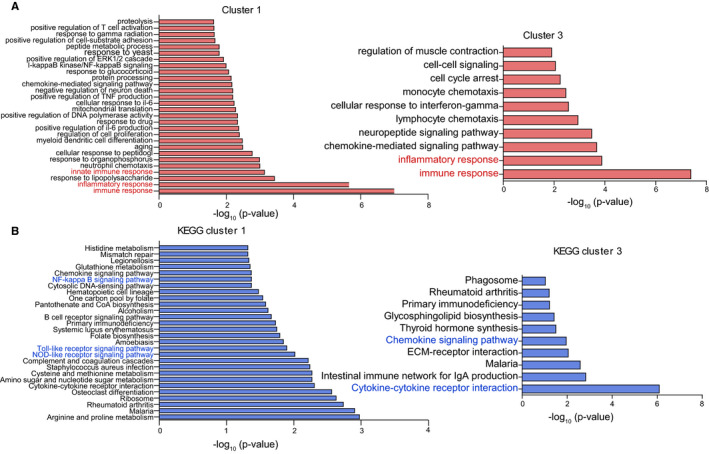
Functional enrichment of differentially expressed genes (DEGs) in response to monocrotaline treatment. A, Gene Ontology (biological process) analysis of the DEGs identified in cluster 1 and cluster 3, biological process terms with only *P*‐value of <.02 and <.01 were showed in cluster 1 and cluster 3, respectively; B, KEGG pathway analysis of the DEGs identified in cluster 1 and cluster 3. KEGG pathway terms with the *P* of <.05 were showed in cluster 1 and cluster 3

Our previous study has showed the elevated markers of macrophage infiltration and inflammatory mediators, such as TNFα and IL‐6, with concomitantly increased pulmonary arterial remodelling parameters WT% and WA% in the progression of MCT‐induced PAH.^7^ RNA‐seq analysis of rat lungs isolated from control and MCT‐treated rats identified a total of 23 200 transcripts, of which 280, 1342, 908 and 3155 were differentially expressed at the end of weeks 1, 2, 3 and 4, respectively.^10^ Further hierarchical clustering analysis of the differentially expressed genes (DEGs) revealed 10 clusters of expression pattern. Cluster 1, cluster 3 and cluster 4 showed an increased pattern. In contrast, cluster 2, cluster 5 and cluster 10 showed the opposite pattern (Figure 1). GO enrichment analysis of all the 10 clusters using DAVID showed that only cluster 1 and cluster 3 whose expression pattern resembled the changes of pulmonary arterial remodelling parameters, WT% and WA%, were associated with inflammatory and immune response (Figure 2A; Figure S1). KEGG pathway enrichment of cluster 1 and cluster 3 using KOBAS revealed 28 significantly enriched pathway terms in cluster 1 and 10 significantly enriched pathway terms in cluster 3. The majority of the pathway terms were linked to inflammation and immunity, including Nod‐like receptor (NLR) signalling pathway, Toll‐like receptor (TLR) signalling pathway and NF‐κB pathway in cluster 1, as well as cytokine‐cytokine receptor interaction and chemokine signalling pathway in cluster 3 (Figure 2B).

### 3.2 Identification of inflammatory and immune profiling

**FIGURE 3:**
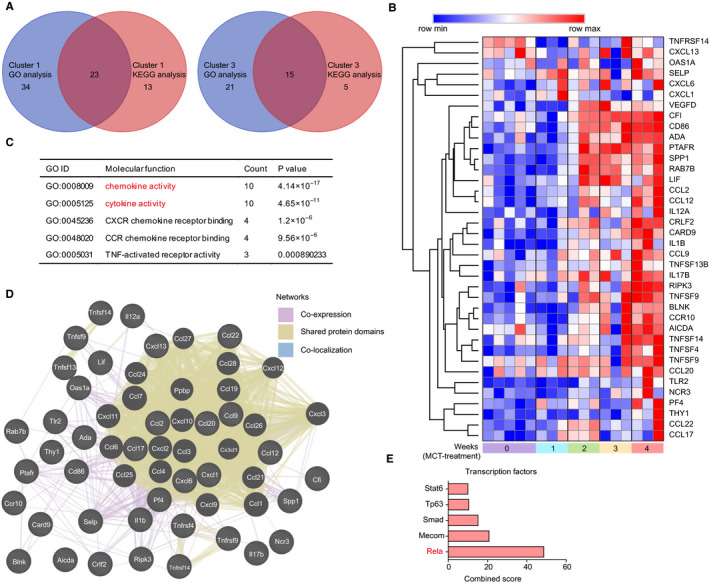
Inflammatory and immune profiling in monocrotaline‐induced pulmonary arterial hypertension. A, Venn diagram showing differentially expressed genes (DEGs) that were overlapped in Gene Ontology (GO) and KEGG enrichment analysis, the overlapped genes was determined by using Venny 2.1 software (https://bioinfogp.cnb.csic.es/tools/venny/). B, Hierarchical clustering of the overlapped DEGs by using Morpheus software (https://software.broadinstitute.org/morpheus/). C, Summary table for overrepresented GO molecular function terms in the overlapped DEGs. D, The network among the overlapped DEGs. E, The enrichment of highly represented transcription factors in overlapped DEGs by using ChEA 2016 database in Enrichr. The significantly enrichment terms were determined by combined score, and the top 5 transcription factors were showed

Further analysis of enriched inflammatory and immune genes in cluster 1 and cluster 3 using Venny 2.1 showed that a total of 70 and 41 genes were linked to inflammation and immunity, of which 23 and 15 were overlapped (Figure 3A). Hierarchical clustering of the overlapped genes using Morpheus showed most of the genes were increased in a time‐dependent manner (Figure 3B). GO enrichment analysis using DAVID showed that the overlapped genes were associated with chemokine and cytokine activity (Figure 3C). Analysis of the interaction among overlapped genes using GeneMANIA revealed that the majority of genes were co‐expressed and shared C‐C/C‐X‐C chemokine domain (Figure 3D). Enrichment analysis using Enrichr revealed that Rela, the p65 subunit of NF‐kB, was the most significantly enriched transcription factor in the overlapped genes (Figure 3E). Collectively, the overlapped genes were co‐expressed, associated with chemokine and cytokine activity and predominantly regulated by NF‐κB pathway, thus maybe representing the inflammatory and immune profiling that lead to pulmonary vascular remoulding in MCT‐induced PAH.

### 3.3 The change of TLR and NLR pathways in MCT‐induced PAH

**FIGURE 4:**
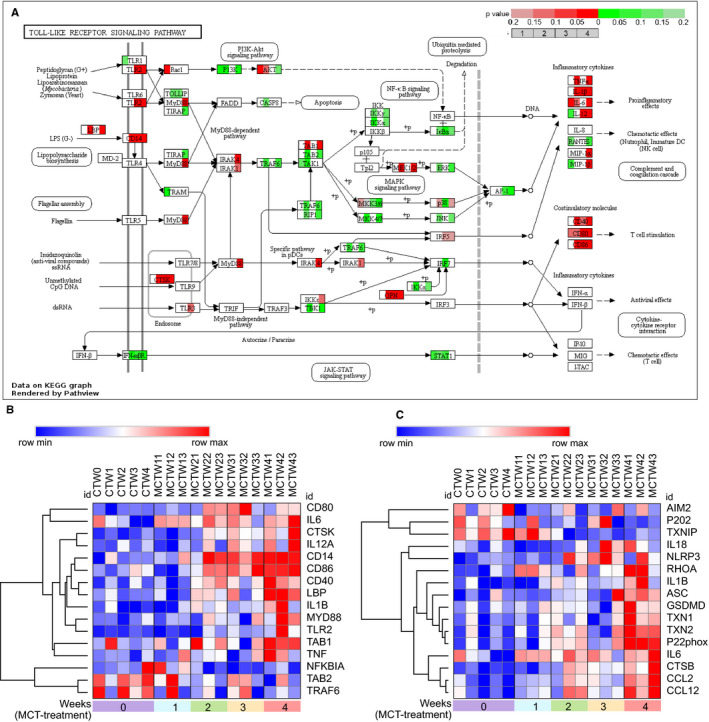
The change of Toll‐like receptor (TLR) and Nod‐like receptor (NLR) pathways in response to monocrotaline (MCT) treatment. A, The integration and visualization of gene expression change in TLR pathways using modified Pathview. Each coloured box represents the comparison of MCT treatment 1 wk with control, MCT treatment 2 wk with control, MCT treatment 3 wk with control and MCT treatment 4 wk with control. Colour represents *P*‐value for each comparison of MCT treatments with control by using Morpheus software (unpaired *t* test); genes with relatively increased and reduced expression were shown in red and green, respectively, while white represents *P* ≥ .2 or not detected. B, C, Heatmap showing differentially expressed genes annotated in TLR and NLR pathways of KEGG database, rows in the heatmap represent gene expression levels, and columns represent each sample

Due to having enrichment of TLR and NLR pathways in cluster 1 by KEGG pathway enrichment analysis, we then characterized the changes of TLR and NLR pathways in response to MCT treatment. The TLRs are specific families of pattern recognition receptors capable of detecting microbes and generating innate immunity. Upon recognizing specific structures of microorganisms, TLRs activate NF‐κB pathway, resulting in the alteration of effector mechanisms, including up‐regulation of TNFα, IL‐1β, IL‐6 and IL‐12.^17^ In addition to showing up‐regulation of previously well‐known effector genes in PAH, such as TNFα, IL‐6 and IL‐12,^2^, ^18^ pathway‐based data integration and visualization using the Pathview and hierarchical clustering analysis of DEGs using Morpheus also revealed up‐regulation of less well‐appreciated genes in TLR pathways, such as up‐regulated genes including TLR2, MyD88, CD14, LBP and TAB1, as well as down‐regulated genes including TAB2, NFKBIA (IκBα) and TRAF6 (Figure 4A,B).

The NLRs were the inflammasomes consisted of an inflammasome sensor such as NLRP3 and AIM2, caspase‐1 and often an adaptor protein ASC. Upon inflammasomes assembly and subsequent caspase‐1 activation, their effector mechanisms were then triggered, including release of activated IL‐1β and IL‐18 and initiation of gasdermin D (GSDMD)–mediated pyroptosis.^19^ In addition to showing up‐regulation of previously reported inflammasome components and their effector molecules including NLRP3, ASC, IL‐1β and IL‐18,^20^ further analysis of NLR signalling pathways using Pathview and Morpheus also showed less well‐characterized genes, such as elevated GSDMD, CTSB, p22phox and TRX1 (TXN1), and reduced AIM2, p202 and TXNIP in MCT‐induced PAH (Figure S2; Figure 4C).

Visualization of global pathway change using Pathview showed that the gene expression changes in TLR and NLR pathways were not always synergistic. As an example, the reduced expression of IκBα, TRAF6 and TAB2 was identified in TLR pathway (Figure 4B). The reduced IκBα, a NF‐κB inhibitor, was associated with activation of NF‐κB pathway. However, the reduced TRAF6 and TAB2 expression may restrict activation of NF‐κB pathway. It was likely that the presence of negative feedback mechanisms prevented the overactivated or prolonged TLR and NLR pathways in the progression of MCT‐induced PAH.

### 3.4 The activation of TLR and NLR pathways by DAMPs

**FIGURE 5:**
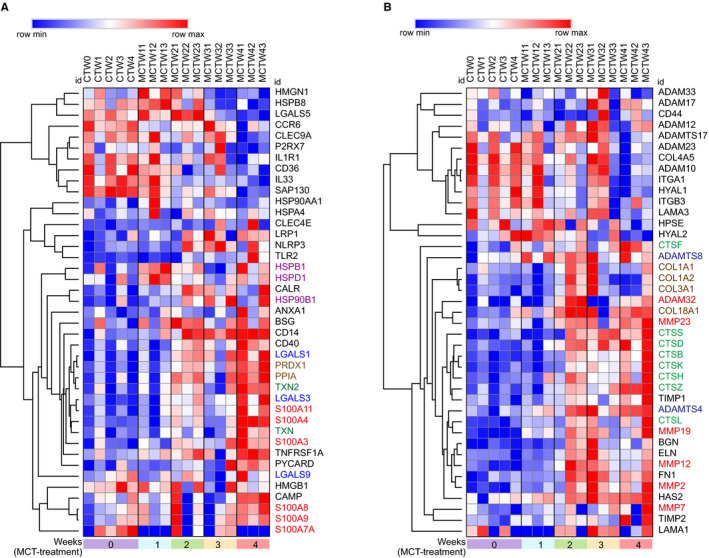
The expression change of damage‐associated molecular patterns (DAMPs) in response to monocrotaline (MCT) treatment. A, Heatmap of differentially expressed intracellular DAMPs induced by MCT. B, Heatmap of differentially expressed extracellular DAMPs and their proteolytic enzymes induced by MCT. CTW, control; MCTW1, MCT treatment for 1 wk; MCTW2, MCT treatment for 2 wk; MCTW3, MCT treatment for 3 wk and MCTW4, MCT treatment for 4 wk

It is well established that TLRs and NLRs could be activated by endogenous molecules termed damage‐associated molecular patterns (DAMPs), and a series of DAMPs and related receptors have been identified by previous studies (Tables S1 and S2). In our RNA‐seq data set, some of the intracellular and extracellular DAMPs were found to be differentially expressed. To characterize these differentially expressed DAMPs in the progression of MCT‐induced PAH, we applied heatmap to exhibit the DEGs of intracellular and extracellular DAMPs, their receptors and proteolytic enzymes responsible for DAMP exposure. Hierarchical clustering of the differentially expressed intracellular DAMPs using Morpheus showed the dysregulated expression of intracellular DAMPs, including elevated expression of galectins (LGALS1 and LGALS1), thioredoxin (TXN and TXN2), S100 proteins (S100A3, S100A4, S100A8 and S100A9 *et al*), cyclophilin A (PPIA), peroxiredoxin 1(PRDX1) and heat shock proteins (HSPB1, HSPD1 and HSP90B1) in MCT‐induced PAH (Figure 5A). In addition, hierarchical clustering analysis of differentially expressed extracellular matrixes (ECMs) also revealed up‐regulation of extracellular DAMPs, including elastin (ELN), biglycan (BGN), collagen components (COL1A1, COL1A2, COL3A1 and COL18A1), fibronectin (FN1) and laminin (LAMA1) (Figure 5B). Notably, the enzymes capable of degrading such ECMs were also elevated, including matrix metalloproteinases (MMP2, MMP7, MMP12, MMP19 and MMP23), cathepsins (CTSB, CTSS and CTSD etc), a disintegrin and metalloproteinases (ADAM32) and a disintegrin and metalloproteinase with thrombospondin motifs (ADAMTS4 and ADAMTS8) (Figure 5B).

### 3.5 The exposure of DAMPs by necroptosis

**FIGURE 6:**
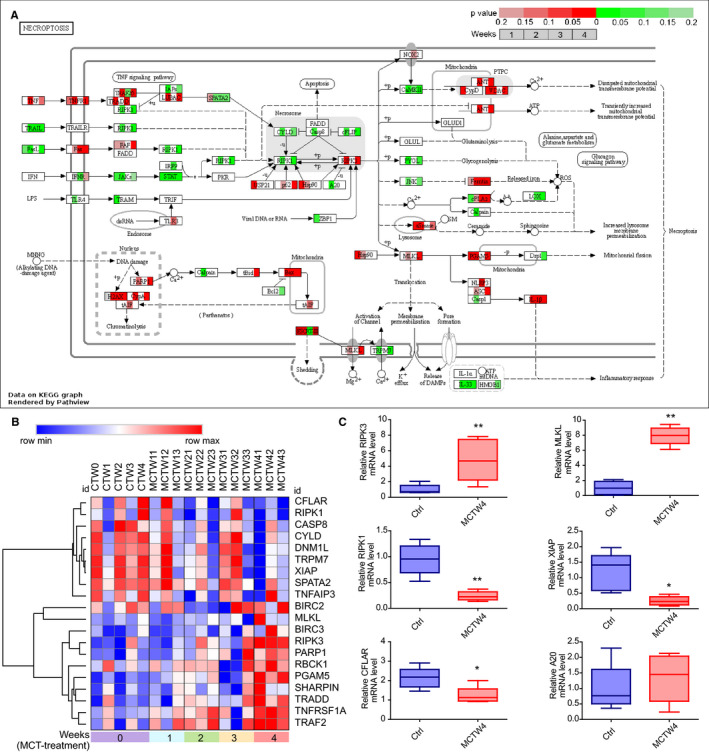
The change of necroptosis pathways in response to monocrotaline (MCT) treatment. A, The integration and visualization of gene expression change in necroptosis pathways using modified Pathview. Each coloured box represents the comparison of MCT treatment 1 wk with control, MCT treatment 2 wk with control, MCT treatment 3 wk with control and MCT treatment 4 wk with control. Colour represents *P*‐value for each comparison of MCT treatments with control by using Morpheus software (unpaired *t* test); genes with relatively increased and reduced expression were shown in red and green, respectively, while white represents *P* ≥ .2 or not detected. B, Heatmap showing differentially expressed genes (DEGs) annotated in necroptosis pathways of KEGG database, rows in the heatmap represent gene expression levels, and columns represent each sample. C, Validation of the expression of critical DEGs annotated in necroptosis pathway by real‐time PCR. Total RNA was extracted from the lung tissues of control and rats injected with MCT for 4 wk. Results are shown as mean ± SEM, (unpaired *t* test, n = 3‐6) The data are presented with box plot histograms and are analyzed by unpaired t test (n=3‐6), *P < .05 vs control, **P < .01 vs control. Ctrl, control; MCTW4, MCT treatment for 4 wk

Receptor interacting protein kinase‐3 (RIPK3)–mediated necroptosis, a regulated necrosis, is recognized to involve in exposure or generation of DAMPs and then elicits inflammation.^21^, ^22^ RIPK3‐mediated necroptosis could be triggered by TNFα signalling,^23^ enhanced by increased activity of RIPK3 and MLKL, as well as by loss of its negative regulators, including RIPK1,^24^, ^25^ XIAP,^26^ A20^27^ and catalytic activity of CFLAR/CASP8 complex.^28^ To determine the role of necroptosis in PAH, we investigated the change of necroptosis pathway in response to MCT treatment. Analysis of necroptosis signalling pathway using Pathview and Morpheus showed increased expression of RIPK3 and MLKL, as well as reduced its negative regulators including RIPK1, XIAP, A20, CASP8 and CFLAR (Figure 6A‐C). Validation of the RNA‐seq data by real‐time PCR confirmed the increased expression of RIPK3 and MLKL, as well as reduced expression of RIPK1, XIAP and CFLAR after MCT treatment for 4 weeks. As a result, these results may suggest that RIPK3‐mediated necroptosis was enhanced in MCT‐induced PAH. Additionally, the elevated expression of TNFα receptor TNFRSF1A and downstream molecules TRADD, TRAF2, RBCK1 and SHARPIN inferred the potential role of TNFα signalling pathway for the necroptosis elicitation (Figure 6A,B).

## 4 DISCUSSION

In the present study, RNA‐seq and bioinformatics methods were used to identify the pathways related to inflammation and immunity in pulmonary vascular remodelling in PAH. We found dysregulated TLR, NLR and necroptosis pathways and a series of elevated DAMPs, as well as an inflammatory and immune profiling that could lead to pulmonary vascular remodelling in MCT‐induced PAH.

It was well established that IL‐1β, IL‐6 and TNF‐α were associated with pulmonary vascular remodelling in PAH.^2^ Consistently, the expression of IL‐1β, IL‐6 and TNF‐α, the NF‐κB target genes, was increased and moreover, an inflammatory and immune profiling was identified in the present study. This profiling was predominantly regulated by the transcription factor, Rela. Rela is also known as the p65 subunit of NF‐kB. Interestingly, Rela has been demonstrated to be the specific member of the NF‐kB family linked to pulmonary vascular remodelling.^29^ Of this inflammatory and immune profiling, it is noteworthy that several inflammatory factors including CCL2,^30^ SELP,^31^ SPP1,^32^ IL17,^33^ IL1B,^2^ IL12A,^2^ ADA^34^ and PF4^35^ have already been demonstrated to be associated with pulmonary vascular remodelling/pulmonary hypertension.

The innate immune system is an evolutionally conserved host defence mechanism against pathogens and innate immune responses are initiated by pattern recognition receptors.^36^ The TLR family is the well‐characterized pattern recognition receptors in terms of recognition of microbial fragments and activation of downstream NF‐kB pathway.^17^ Pathogen recognition by TLRs is linked to a cascade of events, including rapid activation of innate immune response by inducing production of proinflammatory cytokines, such as IL‐1β, IL‐6, IL12 and TNF‐α, as well as up‐regulation of costimulatory molecules CD40, CD80 and CD86. Given the up‐regulation of TLR2 and downstream molecules and in combination of identification of NF‐κB pathway, it could be speculated that activation of TLR2 initiated a cascade, resulting in the activation of downstream NF‐κB pathway in PAH. This speculation could be supported by the studies showing that TLR2 and its gene polymorphism were robustly associated with the increased levels of inflammatory mediators and development of PAH in patients with systemic sclerosis.^37^, ^38^

TLR4 may also be of relevance, due to up‐regulation of its coreceptor CD14 and LBP and downstream molecules. Furthermore, it was reported that genetic deletion of TLR4 attenuated chronic hypoxia‐induced pulmonary hypertension.^18^ TLR4/NF‐κB pathway has been involved in the inflammatory response related to other cardiovascular diseases, such as acute myocardial infarction and targeting this pathway was suggested to offer an effective therapeutic approach to preserve function of ischaemic heart in patients.^39^ Given the similar role of TLRs (TLR2 and TLR4)/NF‐κB pathway in the development of MCT‐induced PAH, inhibiting of TLRs/NF‐κB pathway may also provide potential clinical implications in patients with PAH, including attenuated inflammatory/immune response and pulmonary vascular remodelling.

The expression of IL‐1β, IL‐18, GSDMD and NLRP3 was elevated in NLR pathway, which was consistent with previously reported activation of NLRP3 inflammasome in PAH.^20^ Lysosomal destabilization and CTSB release were capable of activating NLRP3 inflammasome following DAMP phagocytosis.^40^ The up‐regulation of CTSB suggested lysosomal destabilization after DAMP phagocytosis was one of the mechanisms that result in the activation of NLRP3 inflammasome in PAH.

Increased deposition of ECMs in pulmonary arterioles contributes to the progression of PAH, and both inhibition of synthesis and genetic deletion of ECMs reduce pulmonary arterial remodelling.^41^, ^42^ The elevated expression of ECMs, including elastin, biglycan, collagens, fibronectin and laminin, was identified in the present study. However, the proteolytic enzymes capable of degrading such ECMs were also elevated, including MMPs (MMP19, MMP2 and MMP7), ADAMTSs (ADAMTS4 and ADAMTS8) and cathepsins (CTSS, CTSB and CTSD).

It was assumed that up‐regulation of proteolytic enzymes would result in the degradation of the ECMs and consequently, increased release of ECM fragments into the peripheral circulation. Consistent with this notion, circulating degradation products of ECMs including collagens (type XVIII collagen, type I collagen and type III procollagen),^43^, ^44^ hyaluronan^45^ and elastin^46^ were elevated in the peripheral blood and moreover correlated with the disease severity and poor prognosis. It was supposed that increase in proteolytic enzyme activity would lead to the reduction in ECMs and pulmonary vascular remodelling. However, increased activity of proteolytic enzymes, which facilitates the reduction in ECMs, resulted in aggravation of pulmonary vascular remodelling,^45^, ^47^, ^48^, ^49^ and inhibition of proteolytic enzymes prevented pulmonary vascular remodelling.^50^, ^51^, ^52^ One possible explanation for these findings was that the degradation products of ECMs, such as collagens, elastin and hyaluronan, were just the so‐called extracellular DAMPs and served as the ligands for activation of TLRs and NLRP3 inflammasome. Collectively, these findings may suggest a link between degradation products of ECMs and dysregulated inflammation and immunity in pulmonary vascular remodelling of PAH. In addition to extracellular DAMPs, a large number of intracellular DAMPs were identified as differential expression in response to MCT in the present study. Of these intracellular DAMPs, galectins (LGALS1 and LGALS3),^53^, ^54^ thioredoxin (TXN and TXN2),^55^, ^56^ cyclophilin A,^57^ HSPs (HSP90B1 and HSPA4)^58^ and S100 proteins (S100A3 and S100A4)^48^, ^59^ have already been demonstrated to mediate pulmonary vascular remodelling, although they were not known as intracellular DAMPs in the PAH field.

It is noteworthy that in normal physiological conditions, intracellular and extracellular DAMPs are segregated and cannot activate TLR and NLR pathways. Once cell necrosis, intracellular DAMPs would be passively released. By contrast, extracellular DAMPs are generated from the degradation products of ECMs by proteolytic enzymes.^60^ Necroptosis is a regulated form of necrosis, which is associated with generation or exposure of DAMPs.^21^, ^22^ Necroptosis was originally defined as being dependent on the kinase RIPK1; subsequently, RIPK3 was also established to be required for necroptosis.^23^ But now, RIPK1 is known to inhibit necroptosis as a negative regulator.^24^, ^25^ In this study, the reduced expression pattern of RIPK1 was observed, which was completely opposite to the expression pattern of RIPK3 in MCT‐induced PAH. Apart from the RIPK1, XIAP^26^ and CFLAR/CASP8 complex^28^ were also known as the negative regulators of necroptosis. In the present study, the expression of XIAP and CFLAR was down‐regulated in the progression of MCT‐induced PAH. As a result, the up‐regulation of RIPK3 and MLKL and down‐regulation of its negative regulators may indicate enhanced RIPK3‐mediated necroptosis in the development of MCT‐induced PAH. Additionally, the elevated expression of TNFα receptor TNFRSF1A and downstream molecules TRADD, TRAF2, RBCK1 and SHARPIN indicated the potential role of TNFα signalling pathway in the initiation of necroptosis.

**FIGURE 7:**
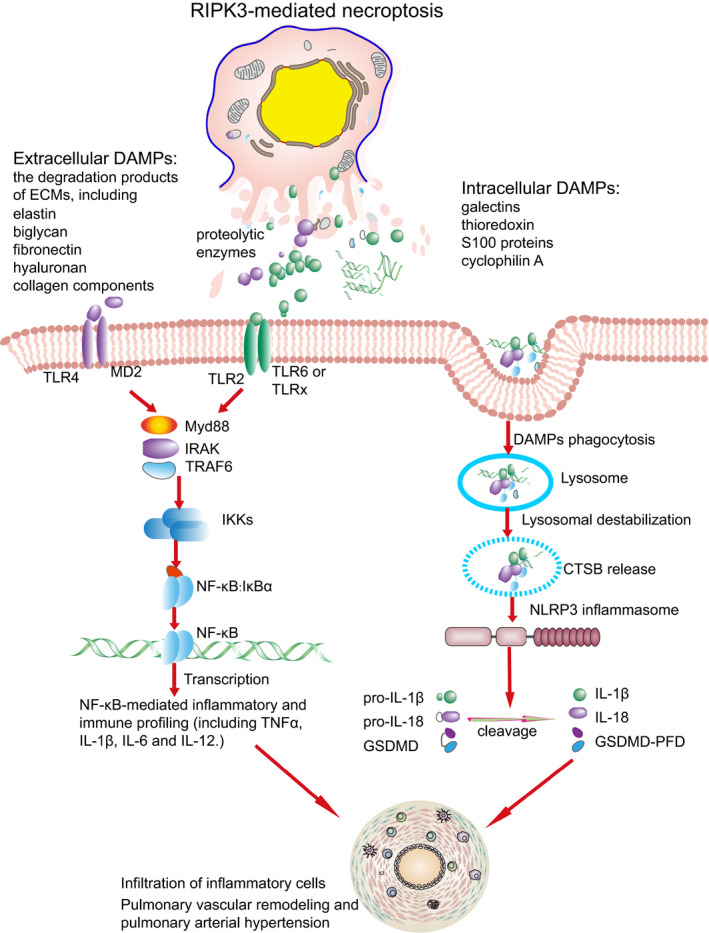
Proposed model outlining a role of Toll‐like receptor (TLR) and Nod‐like receptor (NLR) pathways and necroptosis in pulmonary vascular remodelling. RIPK3‐mediated necroptosis triggered the exposure of intracellular and extracellular damage‐associated molecular patterns and subsequent activation of TLR and NLR pathways. The activated TLR and NLR pathways were responsible for up‐regulation of inflammatory and immune profiling. The up‐regulated inflammatory mediators lead to inflammatory cell infiltration and pulmonary vascular remodelling in pulmonary arterial hypertension

To our knowledge, this is the first time to reveal a role of RIPK3‐mediated necroptosis in the pathogenesis of PAH through bioinformatics analysis. Thus, it is likely that PAH also belongs to the necroptosis diseases. Accordingly, a model illustrating the role of necroptosis and its triggered TLR and NLR pathways in PAH is proposed in Figure 7. This study has some limitations. Firstly, only lung tissues were chosen for gene expression analysis rather than specific cell types. Secondly, the biological replicates in MCT treatment groups may be limited. Finally, this study was based on transcriptomic data, the protein levels, for instance IκBα, IL1β, IL18 and TLR2, and the phosphorylation states of RIPK3 and MLKL proteins were not examined. Consequently, further studies in the future may be needed to verify the role of necroptosis in PAH.

In summary, we identify dysregulated TLR and NLR pathways in the progression of pulmonary vascular remodelling. RIPK3‐mediated necroptosis may be associated with exposure of DAMPs and consequent activation of TLR and NLR pathways. Thus, these results provide novel insights into the mechanisms underlying immunity and inflammation in PAH.

## CONFLICT OF INTEREST

The authors confirm that there are no conflicts of interest.

## AUTHOR CONTRIBUTION

**Genfa Xiao:** Conceptualization (equal); Data curation (equal); Formal analysis (equal); Investigation (lead); Methodology (lead); Writing‐original draft (lead); Writing‐review & editing (equal). **Wei Zhuang:** Resources (equal); Writing‐review & editing (supporting). **Tingjun Wang:** Resources (equal); Writing‐review & editing (supporting). **Guili Lian:** Conceptualization (supporting); Investigation (supporting); Methodology (supporting); Writing‐review & editing (supporting). **Li Luo:** Investigation (supporting); Writing‐review & editing (supporting). **Chaoyi Ye:** Investigation (supporting); Writing‐review & editing (supporting). **Huajun Wang:** Supervision (supporting); Writing‐review & editing (supporting). **Liangdi Xie:** Conceptualization (equal); Funding acquisition (lead); Resources (lead); Supervision (lead); Writing‐review & editing (equal).

## Supporting information

Supplementary Material

## Data Availability

The raw RNA‐seq data that support the findings were deposited in the gene expression omnibus (GEO) repository with an accession number GSE149713.
