# Supplementary material for: Transcriptomic analysis identifies Toll‐like and Nod‐like pathways and necroptosis in pulmonary arterial hypertension
Source: J Cell Mol Med. 2020 Aug 29;24(19):11409–21. doi: 10.1111/jcmm.15745 (PMC7576255; doi:10.1111/jcmm.15745)
Supplement: Supplementary file 1 — Supplementary Material [file JCMM-24-11409-s001.docx]

**Supplemental Materials for**

**Transcriptomic analysis identifies Toll-like and NOD-like pathways and necroptosis in pulmonary arterial hypertension**

Genfa Xiao^1,2,3^, Wei Zhuang^3,4^, Tingjun Wang^1,2,3,4^, Guili Lian^3^, Li Luo^1,2,3^, Chaoyi Ye^3^, Huajun Wang^3^, Liangdi Xie^1,2,3^

^1^ Department of Geriatric Medicine, The First Affiliated Hospital of Fujian Medical University, Fuzhou, People’s Republic of China.

^2^ Department of General Medicine, The First Affiliated Hospital of Fujian Medical University, Fuzhou, People’s Republic of China.

^3^ Fujian Hypertension Research Institute, The First Affiliated Hospital of Fujian Medical University, People’s Republic of China.

^4^ These authors contributed equally to this work.

Correspondence

Liangdi Xie, Department of Geriatric Medicine, The First Affiliated Hospital of Fujian Medical University, No 20 Chazhong Road, Fuzhou 350005, People’s Republic of China.

E-mail address: ldxield@163.com.

**Supplemental figures**

**Supplementary Figure 1.** GO enrichment analysis of DEGs in response to MCT treatment Gene Ontology (biological process) enrichment analysis of the DEGs identified in the clusters, 8 clusters including cluster 2, cluster 4, cluster 5, cluster 6, cluster 7, cluster 8, cluster 9 and cluster 10 were showed; *p* values of <0.05 were regarded as significant and only the top 10 biological process terms were showed in the figures.

**
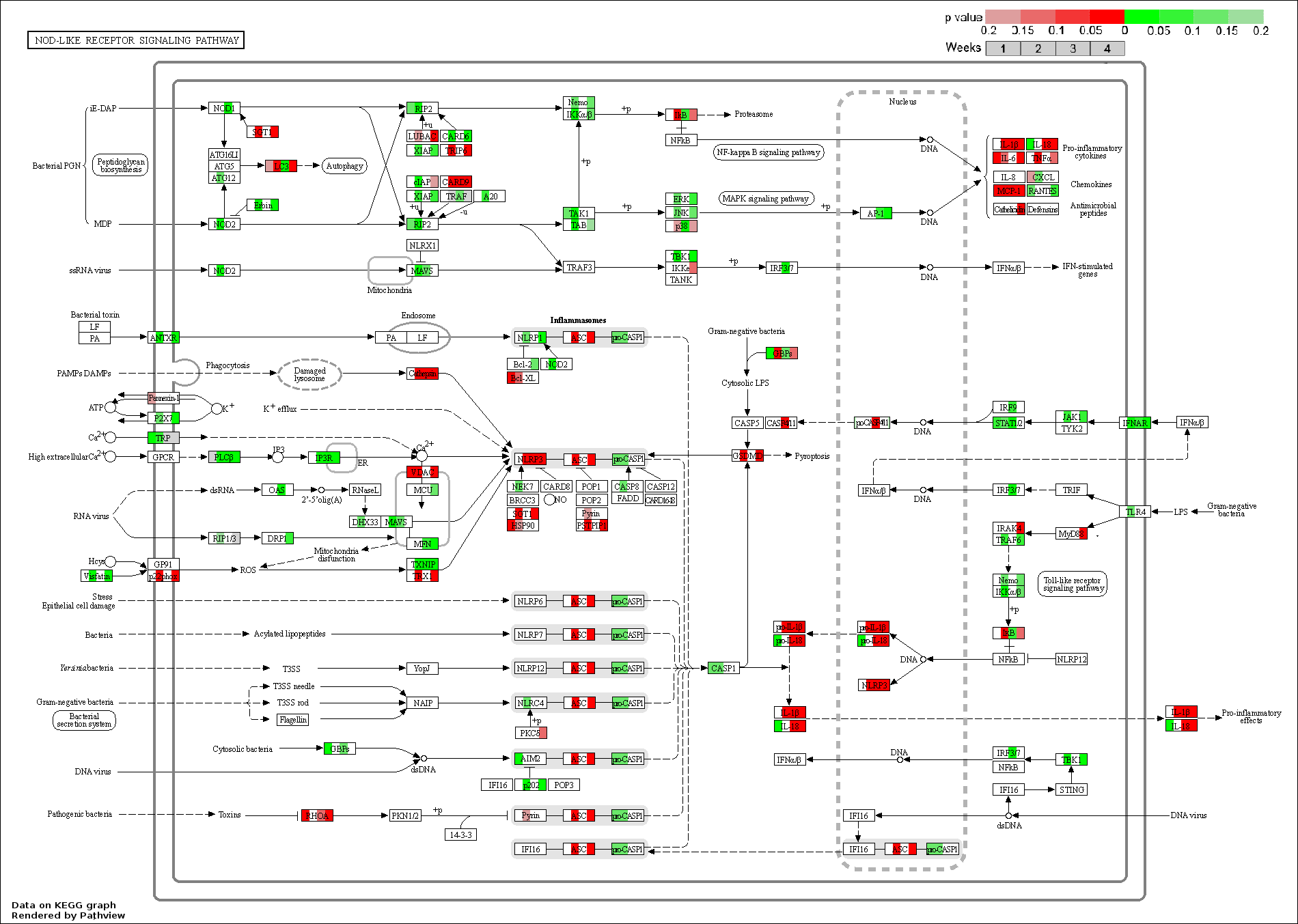
**

**Supplementary Figure 2.** The integration and visualization of gene expression change in NLR pathway using modified Pathview Each colored box represents the comparison of MCT-treatment 1 week with control, MCT-treatment 2 weeks with control, MCT-treatment 3 weeks with control and MCT-treatment 4 weeks with control. Color indicates *p* value for each comparison of MCT-treatments with control by using Morpheus software (unpaired t-test); genes with relatively increased and reduced expression were shown in red and green, respectively, while white represents *p*≥0.2 or not detected.

**Supplemental tables**

**Supplementary table 1. Intracellular DAMPs**

| Intracellular DAMPs | Receptors | Release Mechanism | References |
| --- | --- | --- | --- |
| HMGB1 | TLR2, TLR4, TLR9, RAGE | Apoptosis, necrosis | [^1-4^](#_ENREF_1) |
| S100 proteins | RAGE, TLR4 | Necroptosis | [^5-7^](#_ENREF_5) |
| HSPs | TLR2, TLR4 | Necroptosis | [^8-10^](#_ENREF_8) |
| HMGN1 | TLR4 | Necroptosis | [^3^](#_ENREF_3) |
| IL-1α | IL-1R | Necroptosis  Pyroptosis | [^11^](#_ENREF_11) |
| IL-33 | IL-33R | Necroptosis | [^12^](#_ENREF_12) |
| Peroxiredoxin 1 | TLR4 | Necroptosis | [^13^](#_ENREF_13) |
| Cyclophilin A |  | Necroptosis | [^14^](#_ENREF_14) |
| SAP130 | Mincle | Necroptosis | [^15^](#_ENREF_15) |
| Galectins | CD2 | Cell death | [^16-19^](#_ENREF_16) |
| Thioredoxin |  |  | [^20^](#_ENREF_20)^,^[^21^](#_ENREF_21) |
| Defensins | CCR2, CCR6, TLR4 | Necrosis | [^22-24^](#_ENREF_22) |
| Surfactant protein-A | TLR4 |  | [^25^](#_ENREF_25) |
| ATP | NLRP3, P2X7 | Necroptosis  Pyroptosis | [^26^](#_ENREF_26)^,^[^27^](#_ENREF_27) |
| Uric acid | P2X7, NLRP3 | Cell death | [^28^](#_ENREF_28)^,^[^29^](#_ENREF_29) |
| Cathelicidins | TLR9, FPRL1 |  | [^30^](#_ENREF_30) |

**Supplementary table 2. Extracellular DAMPs**

| Extracellular DAMPs | Potential receptors | Proteolytic enzymes | References |
| --- | --- | --- | --- |
| Biglycan | TLR2, TLR4, NLPR3, P2X7 | MMPs | [^31^](#_ENREF_31)^,^[^32^](#_ENREF_32) |
| Fibrinogen | TLR2, TLR4 |  | [^33^](#_ENREF_33) |
| Fibronectin | TLR2, TLR4 | MMPs | [^34^](#_ENREF_34)^,^[^35^](#_ENREF_35) |
| Aggrecan | TLR2 | MMPs, ADAMTSs | [^36^](#_ENREF_36) |
| Hyaluronan | CD44, TLR2, TLR4, NLRP3 | Hyaluronidase | [^37-39^](#_ENREF_37) |
| Heparan sulfate | TLR4 | Heparanase | [^40^](#_ENREF_40)^,^[^41^](#_ENREF_41) |
| Versican | TLR2, TLR6, CD14 | ADAMTSs | [^42^](#_ENREF_42)^,^[^43^](#_ENREF_43) |
| Collagen | CXCR2 | MMPs | [^44^](#_ENREF_44) |
| Elastin | Integrins | MMPs, CTSS | [^45^](#_ENREF_45)^,^[^46^](#_ENREF_46) |
| Laminin | Integrins | ADAMs | [^47^](#_ENREF_47) |
| Decorin | TLR2, TLR4 | MMPs | [^48^](#_ENREF_48) |

**References**

1. Tian J, Avalos AM, Mao SY, et al. Toll-like receptor 9-dependent activation by DNA-containing immune complexes is mediated by HMGB1 and RAGE. *Nat Immunol*. 2007; 8: 487-96.

2. Yanai H, Ban T, Wang Z, et al. HMGB proteins function as universal sentinels for nucleic-acid-mediated innate immune responses. *Nature*. 2009; 462: 99-103.

3. Yang D, Postnikov YV, Li Y, et al. High-mobility group nucleosome-binding protein 1 acts as an alarmin and is critical for lipopolysaccharide-induced immune responses. *J Exp Med*. 2012; 209: 157-71.

4. Bell CW, Jiang W, Reich CF, 3rd, et al. The extracellular release of HMGB1 during apoptotic cell death. *Am J Physiol Cell Physiol*. 2006; 291: C1318-25.

5. Hofmann MA, Drury S, Fu C, et al. RAGE mediates a novel proinflammatory axis: a central cell surface receptor for S100/calgranulin polypeptides. *Cell*. 1999; 97: 889-901.

6. Tan X, Zheng X, Huang Z, et al. Involvement of S100A8/A9-TLR4-NLRP3 Inflammasome Pathway in Contrast-Induced Acute Kidney Injury. *Cell Physiol Biochem*. 2017; 43: 209-22.

7. Chen B, Miller AL, Rebelatto M, et al. S100A9 induced inflammatory responses are mediated by distinct damage associated molecular patterns (DAMP) receptors in vitro and in vivo. *PLoS One*. 2015; 10: e0115828.

8. Zininga T, Ramatsui L, Shonhai A. Heat Shock Proteins as Immunomodulants. *Molecules*. 2018; 23: E2846.

9. Lehnardt S, Schott E, Trimbuch T, et al. A vicious cycle involving release of heat shock protein 60 from injured cells and activation of toll-like receptor 4 mediates neurodegeneration in the CNS. *J Neurosci*. 2008; 28: 2320-31.

10. Wheeler DS, Chase MA, Senft AP, et al. Extracellular Hsp72, an endogenous DAMP, is released by virally infected airway epithelial cells and activates neutrophils via Toll-like receptor (TLR)-4. *Respir Res*. 2009; 10: 31.

11. England H, Summersgill HR, Edye ME, et al. Release of interleukin-1α or interleukin-1β depends on mechanism of cell death. *J Biol Chem*. 2014; 289: 15942-50.

12. Bonilla WV, Fröhlich A, Senn K, et al. The alarmin interleukin-33 drives protective antiviral CD8⁺ T cell responses. *Science*. 2012; 335: 984-9.

13. Matsumura K, Iwai H. Peroxiredoxin 1 Contributes to Host Defenses against Mycobacterium tuberculosis. *J Immunol*. 2016; 197: 3233-44.

14. Dear JW, Simpson KJ, Nicolai MP, et al. Cyclophilin A is a damage-associated molecular pattern molecule that mediates acetaminophen-induced liver injury. *J Immunol*. 2011; 187: 3347-52.

15. Yamasaki S, Ishikawa E, Sakuma M, et al. Mincle is an ITAM-coupled activating receptor that senses damaged cells. *Nat Immunol*. 2008; 9: 1179-88.

16. Sano H, Hsu DK, Yu L, et al. Human galectin-3 is a novel chemoattractant for monocytes and macrophages. *J Immunol*. 2000; 165: 2156-64.

17. Dai SY, Nakagawa R, Itoh A, et al. Galectin-9 induces maturation of human monocyte-derived dendritic cells. *J Immunol*. 2005; 175: 2974-81.

18. Dapat IC, Pascapurnama DN, Iwasaki H, et al. Secretion of Galectin-9 as a DAMP during Dengue Virus Infection in THP-1 Cells. *Int J Mol Sci*. 2017; 18.

19. Sato S, St-Pierre C, Bhaumik P, et al. Galectins in innate immunity: dual functions of host soluble beta-galactoside-binding lectins as damage-associated molecular patterns (DAMPs) and as receptors for pathogen-associated molecular patterns (PAMPs). *Immunol Rev*. 2009; 230: 172-87.

20. Bertini R, Howard OM, Dong HF, et al. Thioredoxin, a redox enzyme released in infection and inflammation, is a unique chemoattractant for neutrophils, monocytes, and T cells. *J Exp Med*. 1999; 189: 1783-9.

21. Schenk H, Vogt M, Dröge W, et al. Thioredoxin as a potent costimulus of cytokine expression. *J Immunol*. 1996; 156: 765-71.

22. Biragyn A, Ruffini PA, Leifer CA, et al. Toll-like receptor 4-dependent activation of dendritic cells by beta-defensin 2. *Science*. 2002; 298: 1025-9.

23. Biragyn A, Coscia M, Nagashima K, et al. Murine beta-defensin 2 promotes TLR-4/MyD88-mediated and NF-kappaB-dependent atypical death of APCs via activation of TNFR2. *J Leukoc Biol*. 2008; 83: 998-1008.

24. Miles K, Clarke DJ, Lu W, et al. Dying and necrotic neutrophils are anti-inflammatory secondary to the release of alpha-defensins. *J Immunol*. 2009; 183: 2122-32.

25. Guillot L, Balloy V, McCormack FX, et al. Cutting edge: the immunostimulatory activity of the lung surfactant protein-A involves Toll-like receptor 4. *J Immunol*. 2002; 168: 5989-92.

26. Garg AD, Krysko DV, Verfaillie T, et al. A novel pathway combining calreticulin exposure and ATP secretion in immunogenic cancer cell death. *Embo j*. 2012; 31: 1062-79.

27. Iyer SS, Pulskens WP, Sadler JJ, et al. Necrotic cells trigger a sterile inflammatory response through the Nlrp3 inflammasome. *Proc Natl Acad Sci U S A*. 2009; 106: 20388-93.

28. Kono H, Chen CJ, Ontiveros F, et al. Uric acid promotes an acute inflammatory response to sterile cell death in mice. *J Clin Invest*. 2010; 120: 1939-49.

29. Martinon F, Pétrilli V, Mayor A, et al. Gout-associated uric acid crystals activate the NALP3 inflammasome. *Nature*. 2006; 440: 237-41.

30. Lande R, Gregorio J, Facchinetti V, et al. Plasmacytoid dendritic cells sense self-DNA coupled with antimicrobial peptide. *Nature*. 2007; 449: 564-9.

31. Schaefer L, Babelova A, Kiss E, et al. The matrix component biglycan is proinflammatory and signals through Toll-like receptors 4 and 2 in macrophages. *J Clin Invest*. 2005; 115: 2223-33.

32. Babelova A, Moreth K, Tsalastra-Greul W, et al. Biglycan, a danger signal that activates the NLRP3 inflammasome via toll-like and P2X receptors. *J Biol Chem*. 2009; 284: 24035-48.

33. Smiley ST, King JA, Hancock WW. Fibrinogen stimulates macrophage chemokine secretion through toll-like receptor 4. *J Immunol*. 2001; 167: 2887-94.

34. Okamura Y, Watari M, Jerud ES, et al. The extra domain A of fibronectin activates Toll-like receptor 4. *J Biol Chem*. 2001; 276: 10229-33.

35. Lefebvre JS, Lévesque T, Picard S, et al. Extra domain A of fibronectin primes leukotriene biosynthesis and stimulates neutrophil migration through activation of Toll-like receptor 4. *Arthritis Rheum*. 2011; 63: 1527-33.

36. Lees S, Golub SB, Last K, et al. Bioactivity in an Aggrecan 32-mer Fragment Is Mediated via Toll-like Receptor 2. *Arthritis Rheumatol*. 2015; 67: 1240-9.

37. Jiang D, Liang J, Fan J, et al. Regulation of lung injury and repair by Toll-like receptors and hyaluronan. *Nat Med*. 2005; 11: 1173-9.

38. Taylor KR, Yamasaki K, Radek KA, et al. Recognition of hyaluronan released in sterile injury involves a unique receptor complex dependent on Toll-like receptor 4, CD44, and MD-2. *J Biol Chem*. 2007; 282: 18265-75.

39. Yamasaki K, Muto J, Taylor KR, et al. NLRP3/cryopyrin is necessary for interleukin-1beta (IL-1beta) release in response to hyaluronan, an endogenous trigger of inflammation in response to injury. *J Biol Chem*. 2009; 284: 12762-71.

40. Johnson GB, Brunn GJ, Kodaira Y, et al. Receptor-mediated monitoring of tissue well-being via detection of soluble heparan sulfate by Toll-like receptor 4. *J Immunol*. 2002; 168: 5233-9.

41. Goodall KJ, Poon IK, Phipps S, et al. Soluble heparan sulfate fragments generated by heparanase trigger the release of pro-inflammatory cytokines through TLR-4. *PLoS One*. 2014; 9: e109596.

42. Kim S, Takahashi H, Lin WW, et al. Carcinoma-produced factors activate myeloid cells through TLR2 to stimulate metastasis. *Nature*. 2009; 457: 102-6.

43. Wang W, Xu GL, Jia WD, et al. Ligation of TLR2 by versican: a link between inflammation and metastasis. *Arch Med Res*. 2009; 40: 321-3.

44. Weathington NM, van Houwelingen AH, Noerager BD, et al. A novel peptide CXCR ligand derived from extracellular matrix degradation during airway inflammation. *Nat Med*. 2006; 12: 317-23.

45. Houghton AM, Quintero PA, Perkins DL, et al. Elastin fragments drive disease progression in a murine model of emphysema. *J Clin Invest*. 2006; 116: 753-9.

46. Chang CJ, Hsu HC, Ho WJ, et al. Cathepsin S promotes the development of pulmonary arterial hypertension. *Am J Physiol Lung Cell Mol Physiol*. 2019; 317: L1-l13.

47. Adair-Kirk TL, Atkinson JJ, Broekelmann TJ, et al. A site on laminin alpha 5, AQARSAASKVKVSMKF, induces inflammatory cell production of matrix metalloproteinase-9 and chemotaxis. *J Immunol*. 2003; 171: 398-406.

48. Merline R, Moreth K, Beckmann J, et al. Signaling by the matrix proteoglycan decorin controls inflammation and cancer through PDCD4 and MicroRNA-21. *Sci Signal*. 2011; 4: ra75.
